# Hate Speech, Emotions, and Gender Identities: A Study of Social Narratives on Twitter with Trainee Teachers

**DOI:** 10.3390/ijerph18084055

**Published:** 2021-04-12

**Authors:** Delfín Ortega-Sánchez, Joan Pagès Blanch, Jaime Ibáñez Quintana, Esther Sanz de la Cal, Raquel de la Fuente-Anuncibay

**Affiliations:** 1 Department of Specific Didactics, Faculty of Education, University of Burgos, 09001 Burgos, Spain; jibanez@ubu.es (J.I.Q.); esanz@ubu.es (E.S.d.l.C.); 2 Integrated Science Association (ISA), Universal Scientific Education and Research Network (USERN), Tehran 1419733151, Iran; 3 Department of Language, Literature and Social Science Education, Autonomous University of Barcelona, 08193 Bellaterra, Spain; joan.pages@uab.cat; 4 Department of Education Sciences, Faculty of Education, University of Burgos, 09001 Burgos, Spain; raquelfa@ubu.es

**Keywords:** hate speech, gender identities, social narratives, social education, trainee teachers, twitter

## Abstract

The objective of this study is, on the one hand, to analyse emotional responses to the construction of hate speech relating to gender identity on Twitter. On the other hand, the objective is to evaluate the capabilities of trainee primary education teachers at constructing alternative counter-narratives to this socially alive issue, surrounding the approval of the *Ley de Identidad de Género* [Gender Identity Law] in Chile, in 2018. With this two-fold objective in mind, quantitative, descriptive, and inferential analysis and qualitative analysis techniques are all applied. The results inform us of the influence of socially constructed emotions and feelings that are expressed in social narratives. However, the narratives of the participants neither appeared to reach satisfactory levels of reflection on the social issues that stirred their own emotional responses, nor on the conflict between reason and the value judgements that they expressed in the digital debate (counter-narratives). These results point to the need to consider both emotions and feelings, as categories of social analysis, and to reflect on their forms of expression within the framework of education for inclusive democratic citizenship.

## 1. Introduction

One of the most controversial aspects of present-day social media is the digital forum where unreasoned affirmations, scornful of otherness, and social exclusion predominate. Among the most well-known spaces for the construction of these narratives of hate, or hate speech, Twitter is a network of networks of special influence in both formal and non-formal fields of education [1,2,3,4], and in the construction of perceptions of social reality.

In this investigation, the relevance of this network will be studied, in relation to its coverage of issues relating to political interest and civil activism [5,6,7,8,9], and environmental challenges and social problems, such as gender inequalities [10,11,12]. Despite its democratic potential, its functional operation as a social space for the emergence of hate speech against social, cultural, sexual, and religious minorities will also be examined, based on the construction of antagonistic narratives that deny dialogical participation and promote symbolic violence [13].

**Figure 1:**
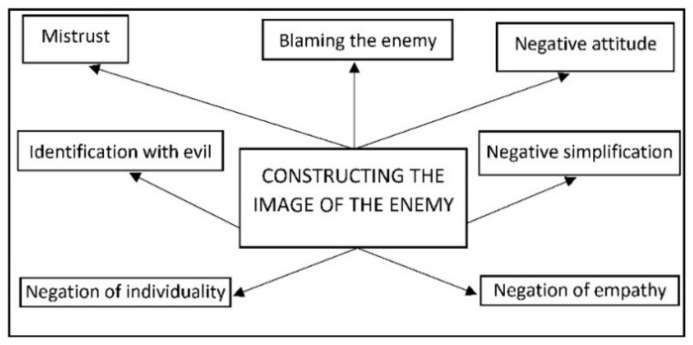
Constructing the image of the enemy [16,17].

The presence of groups in a contextualized situation of vulnerability has to be recognized, and negative and stereotyped intentions, explicitly directed at humiliation and social segregation, have to be identified, in order to consider the existence of hate speech or narratives of hate [14,15]. Their constructive basis is the ‘image of the enemy’ and, in consequence, they are contrary to the idea of diversity (Figure 1).

The European Commission against Racism and Intolerance of the European Council defined hate speech or incitation to hatred as: “*The advocacy, promotion or incitement of the denigration, hatred or vilification of a person or group of persons, as well any harassment, insult, negative stereotyping, stigmatization or threat of such person or persons and any justification of all these forms of expression—that is based on a non-exhaustive list of personal characteristics or status that includes “race”, colour, language, religion or belief, nationality or national or ethnic origin, as well as descent, age, disability, sex, gender, gender identity and sexual orientation*” ([18], pp. 2–3).

In the narratives on hatred, the emotions predominate over and above rationality. In fact, most of their affirmations are not consistent when justified with facts, data, and arguments [19]. Cognitivists, sociologists, and anthropologists have maintained that both emotions and feelings have a direct influence on the following: perceptions of social reality and its future path, communications, personal decision-making, creativity and value systems [20,21,22,23]. In fact, the first large-scale systematic studies on the measurement of hate speech on social media (Whisper and Twitter) and their analysis, covered in the study of Mondal et al. [24], provided evidence of these patterns and their presence on the construction of the narratives.

Working with narratives on hate in the classroom constitutes one of the educational demands of our day and age. The Council of Europe [25] has, on this point, promoted specific actions to combat these sorts of narratives on social media, through the promotion of human rights, democratic participation, and preliminary courses on the critical analysis of the communications media. Teacher-training programmes have to incorporate these digital social narratives or stories, in particular, those who generate hate, to work with them and to offer the necessary tools to counter them with democratic values and the principles of social justice.

The teaching of social sciences must intervene in the training of the critical thought processes of students for the dialectical interpretation of social reality, intervention in social problems [26], and the promotion of alternative narratives to hate speech. The construction of these counter-narratives, alternative stories or narrative forms for the “*development of critical thought oriented towards social action*” ([16], p. 428), ought to “*help to interpret the role of the emotions in hate narratives and to offer information to position reason in opposition to irrationality*” ([16], p. 427), revealing their contradictions and their stereotypes.

In this context, the objective of the present research is to analyse the mediating roles of the emotions, bias, and partiality in digital social narratives [27] in relation to socially alive issues within public debates, in particular, on the Twitter social network. It likewise seeks to evaluate the way in which social sciences trainee-teachers of place reason in opposition to value judgements expressed on Twitter for managing and changing mediating emotions, especially when they are “*negative, perverse, or inconvenient*” ([21], p. 32). From this perspective, the capabilities of trainee-teachers to construct alternative social counter-narratives to digital hate stories concerning gender inequalities will be determined, in the context of the recent approval in Chile of the Gender Identity Law.

This paper is structured in the following sections and subsections: Section 1. Introduction; Subsection 1.1. Influence of emotions in the construction of social narratives and its role in education for democratic and inclusive citizenship; Section 2. Materials and Methods; Subsection 2.1. Design and procedure; Subsection 2.2. Participants; Subsection 2.3. Instrument; Subsection 2.4. Data analysis; Section 3. Results; Subsection 3.1. Quantitative content analysis; Subsection 3.2. Qualitative content analysis; Subsection 3.2.1. First category: beginner; Subsection 3.2.2. Second category: intermediate; Subsection 3.2.3. Third category: expert; Section 4. Discussion; Section 5. Conclusions; Section 6. Limitations.

### 1.1. Influence of Emotions in the Construction of Social Narratives and Its Role in Education for Democratic and Inclusive Citizenship

According to Morgado: “*our social behavior, that is, our entire relationship with other people, is particularly influenced by emotions and feelings*” ([21], p. 32). Emotions, understood as social constructions, constitute, therefore, a valid category of social analysis and object of cultural study [28]. From this perspective, the changing social and cultural nature of the forms of expression of emotions allows that “*they can be harnessed to visualize the historical and social meaning of internal or subjective life*” ([28], p. 189).

Despite the proximity and semantic relationship between emotion and feeling, Damasio [29] identifies the boundary between the two terms in the space, subjective or social, in which they are experienced. Indeed, while feeling starts from the mental and private experience of emotion, emotion represents its manifestation, usually external, observable and, therefore, measurable. Consequently, emotions would be the most recognizable expressions in social interpretation and explanation.

The highlighting of emotions in the teaching of social knowledge and in teacher training renounces the reason-emotion split, characteristic of modern rationality [29], to explain social behavior by integrating the intervention of emotions. Emotions, in fact, “*cohere the feeling of community of individuals and human groups, and are expressed in recognizable gestures*” ([30], p. 357). This emotional and affective cohesion was demonstrated in the use by secondary education students of certain exclusionary political and historical narratives during the Chilean feminist movement in 2018 [31]. Similarly, it could be evidenced in the digital analysis of the #MeToo movement, in which the polarized use of aggressive and destructive language versus more positive ones oriented towards education and the defense of human rights was identified [32].

The incorporation of emotional experience in the training of future teachers, and the analysis of the relationship between emotions and social change [33] become valid categories for the understanding, interpretation and explanation of a naturally plural and diverse social reality. In this sense, working digital critical literacy in the teaching of social sciences implies revealing emotions as stimuli that induce a certain affective sociability. Anger, one of the most recognizable universal primary emotions in public social behavior or hate permeate human history, and thus shape its social narratives [34], especially, social narratives of hate.

Recent research on the LGBT community has focused on the analysis of emotional well-being and the impact of health inequalities, particularly for transgender people. Within the scope of these inequalities, emotions are placed in a central place, whose adversities are found in all spaces of social relations, including the digital, the educational and the health care [35].

The high frequency of use among university students [36,37,38] and adolescents of Twitter for self-expression, communication, information exchange, and the emergent and cooperative preparation of multi-modal narratives [39,40], as well as the positive attitudes of teaching staff towards their communicative potential for professional development have been sufficiently well studied [41,42]. Nevertheless, investigations that are specifically directed toward the analysis of hate speech on the most popular digital media and its effects on people, social groupings, and collectives are scarce, and almost inexistent in the context of Latin-America.

Despite the recognition of the difficulties of detecting hate speech in social networks, there have been significant technological advances in identifying, semantically capturing, and automatically monitoring tweets aimed at constructing this type of speech on Twitter [43,44,45]. However, there has been very little research focused on social science teacher education and the analysis of its implications for democratic citizenship education.

The most recent, from the sociological perspective, may be found in the work of Lingiardi et al. [46], who applied a lexical analysis of semantic context within the framework of the project ‘Map of Italian hate’, with the objective of completing a ‘social snapshot’ of behaviours and social attitudes. Their results confirmed that women and the LGTBIQ collective were found among the most hated groups on Twitter.

On the basis of the above objectives, the following research questions are proposed:

(1) Is there a statistically dependent relation between the sex of the participant and the emotions experienced after reading the digital debate, in Spanish, on the approval of the Chilean Gender Identity Law?
(2) Will significant relations of interdependency be noted between the tweets of the digital debate, the emotions that are experienced, and their textual justifications?
(3) How will trainee-teachers describe their manifest and/or latent emotions? Will they achieve satisfactory levels of reflection and/or justification for the construction of social counter-narratives to hate?

## 2. Materials and Methods

### 2.1. Design and Procedure

The study can be described as a mixed method sequential explanatory design approach [47]. It was planned in two phases: the first directed towards the collection and analysis of quantitative data (from 3 to 5 December 2018), and the second was centred on the collection (from 12 December to 14 December 2018), analysis, description, interpretation, and evaluation of qualitative data. The second phase sought to triangulate and to improve understanding of the quantitative results [48].

The sequential explanatory design (DEXPLIS) is characterized by the integrated follow-up of two research stages: in the first, quantitative data are collected and analyzed and, in the second, qualitative data are collected and interpreted. From this methodological procedure, “*the mixed mixture occurs when the initial quantitative results inform the collection of qualitative data*” ([47], p. 566).

Specifically, our purpose was to use, as a priority, the qualitative results to assist and deepen the explanation of the initial quantitative results. In this sense, DEXPLIS was used in the study to “*characterize cases*” ([47], p. 566) [in our study, a classroom of the last year of the primary education degree], through the features and elements of interest of the proposed research problem.

**Figure 2:**
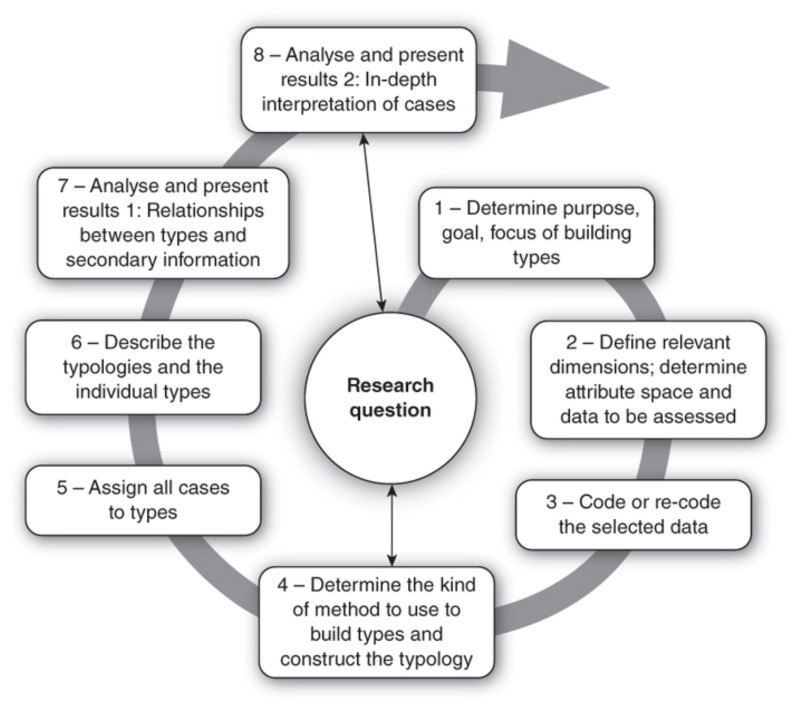
Qualitative research process [49].

A deductive approach to coding and categorization was used, as the intention was to test and extend an existing theory, and to investigate its transferability to a different social context [49]. According to this methodological approach, the following phases were followed, based on the process described in Figure 2: (1) Determination of codes and categories based on the research question. (2) Identification and definition of the textual fragments relevant to our categories. (3) Compilation of all coded segments into categories. (4) Definition of the levels for assessing the relevance of each category (5) Coding of the complete data set. (6) Exhaustive category-based analysis.

Prior to the textual analysis, a provisional code system/book and coding rule (memos) for the identification and segmentation of the qualitative data were developed and its categorization was defined. This deductive coding and categorization derived from the review of previous scientific theory and literature, in particular, the studies of Santisteban et al. [50], and Castellví et al. [51]. Once the coding system was defined and applied to the narratives under study, the three categories (beginner, intermediate and expert), suggested and also applied in the aforementioned studies, were applied according to the level of competence and critical digital literacy of the participating trainee teachers.

Likewise, the case-study methodology was applied [52], with the aim of analysing the capability of a specific group of trainee teachers from a social sciences teacher-training programme on social invisibility and gender identities to construct counter-narratives. This circumstance explains the relevance of its selection and its expected potential informative and interpretative richness. The application of the instrument was completed over the last week of the year in a single 50-min session.

### 2.2. Participants

The total sample consisted of 46 trainee teachers (women: *f_i_* = 34; 73.9%; men: *f_i_* = 12; 26.1%), with an average age of 23.3 years (*SD* = 2.52). Non-probabilistic convenience sampling was applied to the available cases and the possibilities of the researchers to access the field of study [53]. The selection of the participants followed one single intentional criterion: to have enrolled on or to have completed a teacher training course on gender identities, social otherness, and social invisibility in the teaching of social sciences, taught by one of the principal researchers at a university in the north of Spain [54].

The sample presents a characteristic male-female distribution of the university population in the area studied, characterized by a greater presence of women. In this sense, the OECD [55] points out great differences according to the areas, greater in education (81%) with respect to health or social services (72%), these percentages being very similar to other European countries such as Ireland and the United Kingdom.

### 2.3. Instrument

The instrument that was applied consisted of a set of activities, designed using a digital reproduction of a real debate on the approval of the Gender Identity Law in Chile on the social network Twitter. From among the 323 tweets monitored through the API Streaming application of Twitter and posted between 28 November 2018, the date on which the law was enacted, and 2 December 2018, a total of 8 were selected on the basis of their explanatory capacity and the way they summarized the opinions and the value judgements expressed by the users of the social network. The names and the Twitter accounts of those participating in the debate were edited out of the screen shots, to protect their identities. The reading order of the pairs of tweets, presented in Table 1, is a replica of its sequential appearance in the social network at the time of its selection and reproduction in the classroom.

This selection responded to its capacity to gather a greater number of responses and, therefore, to generate debate among the participants in the discussion. In this sense, the number of interventions derived from its publication was considered for their selection. Based on this numerical orientation, in order to analyse the relevance of this selection (outstanding explanatory capacity in the set of tweets collected; ability to generate emotions in readers; and use of clear language in accordance with the emotional message conveyed), we calculated Kendall’s concordance coefficient, based on the ratings expressed by two independent expert-judges in an interval of 1 to 5 points. The results show the existence of a high concordance index and significant homogeneity between the assigned ranges (*W* = 0.812, *p* = 0.000), confirming the validity of the proposed selection.

The first of three activities asked the trainee teachers to select, from among a group of seven primary universal emotions–disgust/repugnance, anger, fear, surprise, happiness, sadness, and apathy- [29], a minimum of one emotion experienced after the complete reading of the digital debate. The second activity, complementary to the first, presented the eight tweets shown above (Table 1), so that the trainee-teachers could relate each one of them with one or various primary emotions. Lastly, the third activity asked the trainee teachers to draft an argumentative and/or justificatory text, of at least 5 lines, on the emotions that they had experienced (activity 1) and those they had related to each tweet (activity 2), which would contain specific references to some of the eight tweets reproduced for the activities.

The objectives of the instrument were related with student participation in the public debates on relevant social problems within the digital media, and with the capabilities of the trainee teachers to interpret them in a critical manner. Its design was therefore based on the need for close examination of the experiences [56] and the emotions that were noted after reading the digital debates, and the use of the results within the teacher training activities of the trainee teachers.

### 2.4. Data Analysis

The quantitative data were processed with both a descriptive (mode and contingency tables of absolute frequencies and relative percentages) and an inferential analysis (*χ*^2^ test, Fisher’s exact test, and Cramer’s V). In the cases where the expected frequencies were less than 5, the Yates correction was applied, interpreting the *p*-value of continuity correction. Likewise, qualitative-quantitative analyses (analysis of simple correspondences) were applied, with a view to describing the existence and the intensity of the relation between the categorical variables included in the instrument. The quantitative data were processed for their treatment and analysis with the SPSS v.25 statistical software package (IBM, New York, NY, USA).

Finally, for the study of the justificatory/argumentative texts of the trainee teachers, the investigation applied content analysis. These qualitative data were coded and categorized a priori. The codes were pre-defined, drawing from the review of the scientific literature [16] and the research questions. Given that the coding was aprioristic, the categories were established before the analysis and the coding was directly applied to the data. The categories were adjusted to the available information, in order to maximize their exclusivity and exhaustiveness and to reach their saturation [57].

In this analysis, two sub-phases were followed: (1) theoretical phase (pre-analytical), in which the textual units (sentences and complete textual segments) were organized and identified; (2) descriptive interpretative (analytical) phase, in which the meaning of the units were coded using the constant comparative method [58,59], and to reduce them for their classification under one of the three pre-defined independent categories (Table 2).

**Figure 3:**
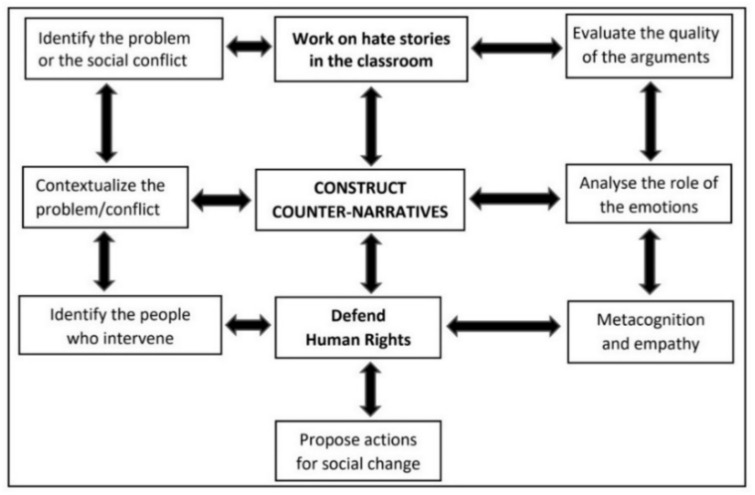
Construct counter-narratives to hate [19].

The analysis was completed in an iterative and recurrent manner, with the purpose of understanding the meaning of the texts written by the trainee teachers from two perspectives: (1) syntactic qualitative-quantitative perspective, through the identification of keywords and their frequency of use in the texts, which explain their manifest and/or latent emotions in relation with the above-mentioned tweets; and, (2) semantic perspective, through the categorical analysis of the narratives [60]. The methodological proposal of Santisteban [19] was used for the interpretation of the discursive arguments and their evaluation, paying special attention to the role of the emotional response and the meta-cognitive and empathic capabilities of the trainee teachers (Figure 3).

The content analysis complied in a satisfactory manner with the qualitative quality criteria: Confirmability (objectivity), transferability (applicability) and representativeness: the data that were collected reached their discursive saturation and their results were audited by an external researcher, which guaranteed their consistency. The content of the recording units was validated by confirming its possible assignment to a single category of analysis, the existence of a clear explanatory capacity of the categories for each of the study codes that made them up (content validity), and the degree of semantic similarity between the coding units and its categorical classification (semantic validity). Likewise, the empirical categories of the study were confirmed in accordance with the research objectives. In addition to checking the appropriateness of the proposed categorical system (validity), the consistency of the measurement procedure (reliability) was also checked. These results will be illustrated with literal fragments highlighted under each corresponding category. Credibility, dependency and trustworthiness: the consensus over the protocol and information collection procedure, and the consensus over the explanatory value of the categories and the coding are a consequence of the agreement between the members of the research team. Procedural reliability in the coding and categorisation of qualitative data was ensured by obtaining a high degree of inter-coder agreement (research team -coder 1- and external researcher -coder 2-) in relation to the criteria of stability (absence of ambiguity in the assignment of recording units to a code and category) and reproducibility (inter-researcher agreement in narrative coding) [61]. The reliability of coding and categorisation [62] was also confirmed by applying Scott’s π index (*π* = 0.82). Atlas.ti software was used for the segmentation of the documents into units of analysis and for their coding, categorization, and textual accountability.

## 3. Results

### 3.1. Quantitative Content Analysis

The emotions that the trainee teachers experienced with greater frequency after reading the digital debate (activity 1) were anger, surprise, and happiness (Table 3).

When these frequencies were analysed by the sex of the trainee teachers, percentile differences were observed between men and women. In fact, while the vast majority of the men chose the emotion of disgust/repugnance (58.3%), surprise (83.3%) and happiness (66.7%) after reading the digital debate, the women selected anger (52.9%) and happiness (76.5%) as their two most representative emotions (Table 4).

These differences were partially confirmed through the existence of statistically significant relations between the sex of the participant and the primary emotions of disgust/repugnance (χ^2^_(1, *n* = 46)_ = 19.09, *p* < 0.001; *F*, *p* < 0.001), and surprise (χ^2^_(1, *n* = 46)_ = 6.31, *p* < 0.05; *F*, *p* < 0.05), two emotions linked, above all, with the men. Likewise, the existence of a significative relation was confirmed between the variable apathy and the sex of the trainee teachers (χ^2^_(1, *n* = 46)_ = 8.56, *p* = < 0.01; *F*, *p* < 0.01), an emotion that was aroused among 33.3% of the men and among 0.0% of the women. These relations registered a high intensity of association with regard to the first emotion mentioned above -disgust/repugnance- (Cramer’s V = 0.713, *p* < 0.001), a low intensity of association with regard to the second emotion −surprise− (Cramer’s V = 0.371, *p* < 0.05) and a medium-moderate intensity of association with the third emotion -apathy- (Cramer’s V = 0.519, *p* < 0.01). No relations of dependency were noted, in contrast, between sex and the emotional variables of anger (*p* ≥ 0.861), fear (*p* ≥ 0.929), happiness (*p* ≥ 0.703), and sadness (*p* ≥ 0.292) (Table 5).

It may be concluded, in consequence, that significative relations of dependency existed between the sex of the participant and the emotions aroused by reading the digital debate on the approval of the Chilean Gender Identity Law (Research question 1). The emotional responses of disgust/repugnance, surprise, and apathy, recorded higher percentile frequencies among the men, while the emotions of anger and happiness were more frequent, although in a statistically non-significative way, among the women.

With a view to obtaining two-dimensional graphic representations of interdependency, by proximity, between the eight tweets, and the emotions they aroused (activity 2) and their textual justifications (activity 3), two analyses were performed between those two variables (χ^2^ = 228.84, *p* < 0.001, accumulated inertia = 81%; χ^2^ = 879.19, *p* < 0.001, accumulated inertia = 58%).

**Figure 4:**
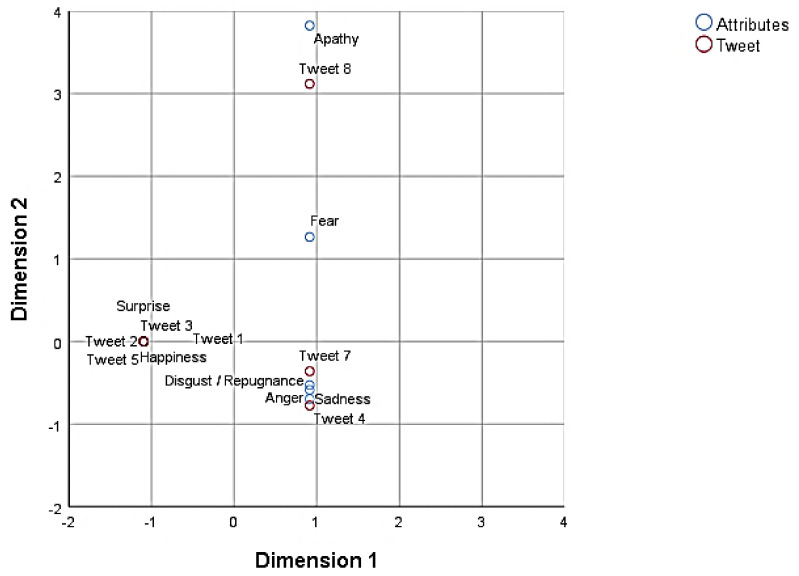
Positions of attributes (emotional variables) and tweets.

As may be seen from the perceptual map (Figure 4), the emotions of surprise and happiness were associated with tweets 1, 2, 3 and 5, those of disgust/repugnance, sadness and anger with tweets 4 and 7, fear with tweets 7 and 8, and apathy with tweet 8. It may therefore be affirmed that the reading of tweets 4 and 7, especially significative for the generation of social narratives of hate, arose from the experience of negative feelings among the trainee teachers.

The absolute frequencies of the attributes associated with the eight tweets are detailed in the table of correspondences (Table 6).

**Figure 5:**
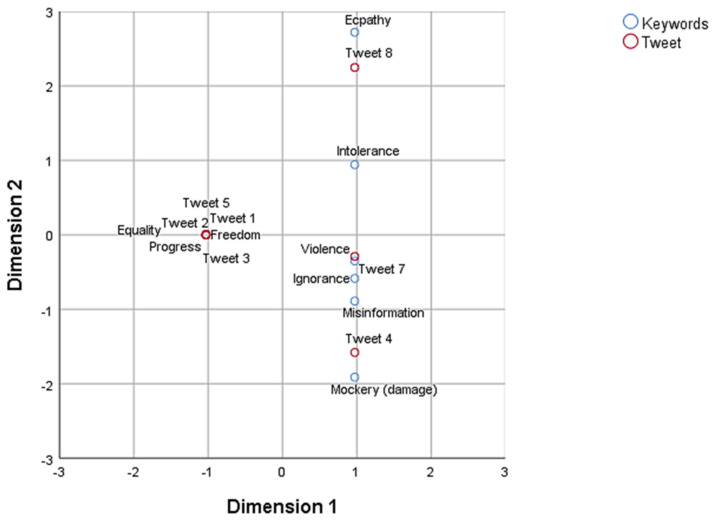
Positions of keywords and tweets.

A total of 43 key words were identified which constituted the most frequently used semantic textual units from the student narratives (activity 3). From this set, the words with a minimum recurrent frequency of five usages were analysed. Perceptual Figure 5 displays the grouping of the keywords ‘freedom’, ‘progress’, and ‘equality’, in relation to *tweets* 1, 2, 3 and 5; the association of the words ‘scorn (harm)’, ‘misinformation’, ‘ignorance’, and ‘violence’ with tweets 4 and 7; and, the absence of ‘empathy’ and the proximity to ‘intolerance’ of tweet 8 (Figure 5). These associations defined and confirmed the general relations that are established in activity 2 (Figure 4).

The absolute frequencies of all the keywords included in the justificatory texts of the trainee teachers are detailed in the corresponding table (Table 7).

The relations represented above offer a response to the second question of the investigation, through which the existence of significative relations of interdependence may be noted between the tweets from the digital debate, the emotions that were experienced and the student narratives. In this sense, the associations of specific negative emotions (disgust/repugnance, sadness, and anger) are evident, and their justifications (‘scorn (harm)’, ‘misinformation’, ‘ignorance’, and ‘violence’) with tweets oriented toward the generation of hate stories (tweets 4 and 7).

### 3.2. Qualitative Content Analysis

The results will be presented below, beginning with the three central aprioristic categories and the most significant textual units from the 302 text excerpts.

#### 3.2.1. First Category: Beginner

The narratives of the trainee teachers that describe the emotions that were selected and related with the eight tweets are contained in the first category (121 excerpts of text). These descriptions reproduce their positions toward the digital debate. However, they were not accompanied by the social, historic, or educational argumentation needed to support their attitudes.

*I feel happiness because, finally, a law has been enacted that recognizes the right to equality and that rejects intolerance of gender diversities (tweets 1, 3 and 6)* […]

[DOA-Bg_(12)_].

*It makes me feel angry, the fact that there are people, self-defined as normal, who have no empathy with those suffering from internal conflicts, because of a socially assigned gender, or that connect their intolerance toward gender diversity with an assumption that children of both sexes have an obligation to change their biological sex (tweets 7 y 8)* […]

[DOA-Bg_(15)_].

*I am happy that this law of gender identity has been approved, in which everybody can feel free. I don’t understand the hurtful and harmful commentaries of some people participating in the debate (tweet 4)*

[DOA-Bg_(22)_].

#### 3.2.2. Second Category: Intermediate

The second category (173 text excerpts) includes the narratives that, although they integrate an acceptable justification for the emotional response that was selected and/or are related with the tweets, contained no explicit reference to hate, so as to reflect on its social and educational causes and consequences.

[…] *It makes me feel happy that part of the population welcomes this sort of progress with enthusiasm. It is clear that they recognize the right to the freedom of people that do not feel the gender [roles] that are socially recognized (tweets 1, 2 and 5)* […]

[JOA-Bg_(24)_; A-RDO-Bg_(24)_; A-RDO-Bg_(24)_].

*It disturbs me that society is so divided over a social problem that is as high-profile as this one. It also makes me indignant that people scorn it and joke about a problem that seriously affects the people that are affected and their environment (tweet 4). It is a revindication of freedom (tweet 1) and, as such, should be valued and respected*

[JOA-Bg_(30)_; A-RDO-Bg_(30)_; A-RDO-Bg_(30)_].

*I am happy to know that there are laws that protect people that need to define their identity at a legal level. This shows that a part of the world is progressing (tweet 2). Egalitarian life is fundamental to democratic citizenship (tweet 3). Why use violence to attack a basic right? (tweet 7)* […]

[JOA-Bg_(3)_; A-RDO-Bg_(3)_; A-RDO-Bg_(3)_].

#### 3.2.3. Third Category: Expert

The third category (eight text excerpts) reunites the units of text with narratives that justify the emotional affirmations that are expressed, reflecting on the causes and the consequences of the social narratives of hate, and evidence capacity for empathy:

*It is sad and it’s not new. We cannot let intolerance (tweet 7) and the lack of empathy (tweet 8) continue limiting social freedom today. Consider that these people speak of ignorance and misinformation (tweets 4 and 7). That’s the reason why I understand that they deny the social reality. Protection of everybody’s rights from all areas of social life should be normalized (tweet 7)*

[JOA-Ex_(35)_; RDO-Ex_(35)_; RCC-Ex_(35)_; E-Ex_(35)_].

*I feel surprise and, at the same time, concern for the people who, in my opinion, do not understand the relevance of the legal recognition of any right. The causes can be found, precisely, in their ignorance (tweets 4 and 7). The key is found in education*

[JOA-Ex_(41)_; RDO-Ex_(41)_; RCC-Ex_(41)_; E-Ex_(41)_].

*Valuing and protecting gender diversity forms part of any inclusive society, which responds to the rights of everybody. I feel fear and concern, because of the open publication of harmful comments (tweet 4) which can arise from attitudes of bullying and in violence against trans or non-binary gender [identities] (tweet 7). These sorts of attacks indicate that a lot of progress is needed. The richness of diversity must be understood through education, and being very aware of signs of intolerance (tweet 7 and tweet 8). […] Hate is not fought with more hate, but with education*

[JOA-Ex_(11)_; RDO-Ex_(11)_; RCC-Ex_(11)_; E-Ex_(11)_].

In accordance with these results, it can be confirmed that the trainee teachers tended to describe and to justify the emotions that they felt, without reaching satisfactory levels of reflection that are sufficient for the integral construction of social counter-narratives to hate (research question 3).

## 4. Discussion

The results that have been obtained have provided information on the influence of emotions and feelings that are socially constructed within the articulation of digital social narratives. Although the trainee teachers coincided in their perceptions of hate speech as obstacles to education for democratic and inclusive citizenship, their narratives neither appeared to reach satisfactory levels of reflection on the social issues that stirred their emotions, nor did they appear to situate reason alongside the value judgements expressed in the digital debate (counter-stories). Our results coincided with those of previous studies [16] uncovering the minority presence of narratives that were capable of rationalizing the incoherencies of hate stories, and providing evidence of the argumentative weaknesses of hate stories, through the emotional responses of the trainee teachers that they themselves recognized and felt. Likewise, the differences identified between the sex and the emotions of trainee teachers are found in the context of earlier works, in which the existence of emotional divergencies are confirmed, as a function of sex, in the contributions to the digital debates on Twitter [63].

Gender differences in relation to socioemotional expressions and self-regulation in educational contexts have recently been demonstrated by Veijalainen et al. [64]. Their results showed the existence of differential emotions in girls and boys in kindergarten; while girls showed emotional expressions related to calmness and peace, boys showed expressions related to surprise, anger or frustration. These differences are likewise evident in the study by Lambrecht et al. [65], whose results report differences in emotion recognition as a function of gender.

Despite the signing of the Code of Conduct on the regulation of hate speech between YouTube, Facebook, and Twitter, and the European Commission [66], we are in agreement with Lingiardi et al. [46] on the urgent need to agree on strategies and to develop preventive campaigns for the eradication of narratives against minority, ethnic, sexual, and gender-specific social groups. Likewise, we coincide with Enarsson and Lindgren [67], in that the presence of these sorts of narratives appears to expand when they form part of a political discussion. In fact, these open narratives operate as a social pressure valve, which contributes nothing to the treatment of and/or the solutions to constructive conflicts that seek to achieve justice and social equality [68].

In agreement with Journell et al. [69], the analyses that have been completed explain the didactic potential of social networks for the acquisition of political commitment by the university students. Likewise, we agree with Dewberry et al. [70] on the need to work with freedom of expression through democratic principles and values within the classroom. From this perspective, the results endorse the need to prepare trainee teachers for education concerning social problems, socially alive issues, and controversial topics within divided societies, considering the emotional variable as one of the most influential factors in their didactic treatment [71]. In this sense, Arneback [72] proposed the application of ‘moral imagination’ in education, which is to say, dwelling on the contextual elements of the social conflict, before deciding on forms of action. From this perspective, this concept has recently been applied to student teachers in order to analyze its influence on the discursive construction of their narratives on ethical dilemmas [73].

Considering emotions and feelings as categories of social analysis and working on their forms of expression constitutes one of the first procedures for education on the construction of counter-narratives to hate. This education would have to start with the identification and the contextualization of the conflict in question, and with the analysis of its origins, causes and up-dating, to continue with the critical evaluation of the emotions that intervene in the rationality of its argumentation. In second place, the acquisition and the evaluation of meta-cognitive capabilities and empathic competences by the trainee teachers would have to become an objective, whilst also considering the emotions that are aroused in them. These competences, essential to conflict resolution and in the development of critical thought, are of special importance in the treatment of social problems for the teaching of Social Sciences [74].

Education in the construction of counter-narratives to hate, as Davids [75] proposed, also implies inquiring into the limits of the forms of ‘tolerable narratives’ within democratic contexts and the legitimacy of the right to the expression of divergent points of view, but contrary to liberty. Hatfield et al. [76] provided one of the keys when defending the promotion of dialogical analysis to confront hate speech and to drive change within higher education.

Teaching staff have to be familiar with these narratives and work with them in the classroom, with the purpose of fighting them in dialectic readings of social reality through the construction of divergent stories (counter-narratives). Education for a democratic citizenship, the ultimate aim in the teaching of social sciences [77], contributes the methodological concepts and the resources that are needed for the deconstruction and for the disclosure of the contradictions of hate speech, and for the construction of counter-narratives based on social justice and human rights [50].

## 5. Conclusions

The relevance of proposing narratives at school that are different from those constructed by curricular materials or the mass media would start, in the first instance, from the identification and recognition of the mediating action of emotions in the construction of social narratives and, in particular, of hate speech. Only in this sense, it would be possible to oppose reason to the deconstruction of social narratives transmitted in school from the theoretical principles of education for a democratic, plural and inclusive citizenship, recognizing, consequently, the bio-cultural status of the human being [78]. In this sense, there are educational initiatives aimed at seeking curricular meeting points between the advances promoted by the Gay-Straight Alliance—Gender-Sexuality Alliance (GSA) and social studies education. Results obtained by Mayo [79] report the advantages of these intersections in promoting change in social education and in eradicating a hidden curriculum, gender differences in academic achievement [80] and “gender-blindness” [81].

The results obtained in the present research highlight the need to revitalize the use of critical discourses [82] on the construction of gender identities in social education. This revitalization would involve knowing how to identify the emotional mediating effects in the construction of social narratives and questioning the impact of hegemonic discourses on gender. The theoretical bases of these critical discourses would have to incorporate, at the very least, feminist practices [83], still with little relevance in Spanish curricula for the training of future social science teachers.

The systemic and structural absence of gender diversities in the social studies curriculum in Spain demands an urgent formative incorporation of the constructive mechanisms of digital discourses. Likewise, considering the differences by gender found in this study, from teacher training it is necessary to reveal the ways in which differential gender socialization occurs, in order to evaluate specific indicators on its consequences on community well-being and the creation of healthy environments [84].

## 6. Limitations

Despite the limitation of the sample size used for this study, due to the type of convenience sampling, its results can be useful for implementing teacher-training programmes on the comprehension and the interpretation of the constructive mechanisms of hate speech in the communication media and social networks, and the construction of alternative social counter-narratives in specific teacher-training contexts. The explanation for this limitation lies in the fact that the research assumes the characteristics of a case study. For this reason, a non-probabilistic convenience sampling (a type of sampling that does not seek a probabilistic representativeness) is applied.

Although the results obtained could differ if the selected participants had been active teachers, our interest was to approach the skills and competencies of trainee teachers who complete their training stage to manage their emotions in their potential participation in digital hate speech. This interest is proposed with the aim of implementing training programs aimed at using these discourses in the construction of counter-narratives from the principles of education for a democratic, plural and inclusive citizenship around gender.

Also, the emotions expressed by the trainee teachers in each of the activities proposed in the classroom constitute a large corpus of self-reported data. This circumstance could lead to biases when writing their activities and to social desirability in the argumentative orientation of their replies.

Although the social network Twitter is not the most popular social network among adolescents, we decided to analyse this digital space in view of the average age of the trainee teachers (23.3 years old), and of the consideration that all participants reported having an active account on this social network.

## Figures and Tables

**Table 1:** Selection of tweets.

RdOrd	Tweets	
1–2	Today the gender identity law was passed, after over 5 years of lobbying by @IgualesChile and @OTDChile. It will mean a substantive improvement in the field of Human Rights in Chile that faces great challenges in the future, especially with respect to children. [Photo: National ID Card My Identity/My Right]	Thanks to those who supported and backed the Gender Identity Law! With the work that we have done together, Chile is a fairer and more egalitarian country. [Photo: Thanks to those who presented the motion on the Gender Identity Law; Also to those who supported it on its way.]
3–4	Historic day. Today the Gender Identity Law has been enacted. We dedicate this day to the victims of transphobia, to those who were murdered for holding an alternative gender identity and who died with an ID card that did not represent them. So that it may never happen again! #HappyWednesday [Photo: Historic Day! Today the Gender Identity Law was enacted. We dedicate this historic day to the 17 fatal victims of transphobia.	Jack can now be a lamp thanks to the Gender Identity Law. [Photo: BIOLOGICALLY I WAS BORN A DOG. But thanks to gender ideology, I do in fact exist! I CAN NOW BE A LAMP if you don’t agree you’re cursed with dog-phobia, living in the past… yours faithfully Progressive Logic]. 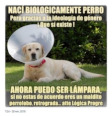
5–6	After years of work and struggle by the trans and LGTB organizations of Chile, Sebastián Piñera approved the new gender identity law, which depathologizes trans identities.	A good day for Chile! The Gender Identity Law was enacted, despite the hysteric opposition (and with no justification) of the religious ultra-right. Common sense and the common good have prevailed, and the guarantee of civil rights and the protection of the most vulnerable.
7–8	Gender identity law, you won’t touch our children with your twisted and fallacious concepts. [Photo: Speech bubbles Adult: “You mustn’t let anybody influence your decision”. Boy: “But I want to be a boy”. Adult: “Shut up you oppressive macho pig! Don’t…”] 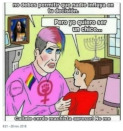	Today is a great day, with the gender identity law an important problem has been solved that affects 0.01% of Chileans. Hopefully as from tomorrow the government will start to concern itself as much with the remaining 99.9%

RdOrd.: Reading Order.

**Table 2:** System of categories.

*C*.	Code	Indicators	Descriptor
Expert	JOA-Ex_(n°-r)_	■ Justification of the chosen emotional response.	Justifies the emotional response with a reasoned argument, evaluates some weaknesses of hate speech and reflects on its causes and consequences. Includes other options and other perspectives and demonstrates a capacity for empathy.
	RDO-Ex_(n°-r)_	■ Reference to hate speech.	
	RCC-Ex_(n°-r)_	■ Reflection on causes and consequences.	
	E-Ex_(n°-r)_	■ Empathy	
Intermediate	JOA-Int_(n°-r)_	■ Justification of the chosen emotional option.	Justifies the emotional response with arguments that reflect the relevant feelings, but makes no explicit reference to hate narratives, with a view to reflecting on the causes and the consequences of the emotion that is aroused.
	A-RDO-Int_(n° -r)_	■ Absence of references to hate stories.	
	A-RCC-Int_(n°-r)_	■ Absence of reflection on causes and consequences.	
Beginner	DOA-Bg_(n°-r)_	■ Description of the chosen emotional option.	In no way justifies the emotional response. Reproduces arguments that only describe the chosen option.

*C*. Category; N°.-r.: number of records.

**Table 3:** Descriptive statistics by emotional variable.

Emotion	*M_o_*_(*n* = 46)_
Disgust/repugnance	2
Anger	1
Fear	2
Surprise	1
Happiness	1
Sadness	2
Apathy	2

1 = Selection of the emotional option; 2 = Absence of any selection of the emotional option.

**Table 4:** Contingency table for the two variables: emotion and sex.

Emotional Variable		Men	Women	Total
Disgust-repugnance	*f_i_*	7	0	7
	*p_i_*	58.3	0.0	15.2
Anger	*f_i_*	6	18	24
	*p_i_*	50.0	52.9	52.2
Fear	*f_i_*	2	8	10
	*p_i_*	16.7	23.5	21.7
Surprise	*f_i_*	10	14	24
	*p_i_*	83.3	41.2	52.2
Happiness	*f_i_*	8	26	34
	*p_i_*	66.7	76.5	73.9
Sadness	*f_i_*	6	10	16
	*p_i_*	50.0	29.4	34.8
Apathy	*f_i_*	4	0	4
	*p_i_*	33.3	0.0	8.7

*fi*: absolute frequency; *pi*: relative percentage.

**Table 5:** Relation and degree of association between the emotional variables and sex.

Emotional Variable	χ^2^	*gl*	*p*	*p-F*	*V*	*p*
Disgust-repugnance	19.090 **	1	0.000	0.000 **	0.713 **	0.000
Anger	0.031	1	0.861	1	0.026	0.861
Fear	0.008	1	0.929	1	0.073	0.620
Surprise	6.317 *	1	0.012	0.018 *	0.371 *	0.012
Happiness	0.080	1	0.777	0.703	0.098	0.506
Sadness	0.874	1	0.350	0.292	0.190	0.198
Apathy	8.569 **	1	0.003	0.003 **	0.519 **	0.000

* *p* < 0.05; ** *p* < 0.01; *p-F*: Fisher’s exact test.

**Table 6:** Table of correspondences of absolute frequencies.

Attributes	Tweets								
	Tweet 1	Tweet 2	Tweet 3	Tweet 4	Tweet 5	Tweet 6	Tweet 7	Tweet 8	Active Margin
Disgust-repugnance				2			5		7
Anger				4			20		24
Fear							6	4	10
Surprise	6	7	4			4			21
Happiness	11	5	6		8				30
Sadness				8			8		16
Apathy								4	4
Active margin	17	12	10	14	8	4	39	8	112

**Table 7:** Table of correspondences of absolute frequencies.

Keywords	Tweet								
	Tweet 1	Tweet 2	Tweet 3	Tweet 4	Tweet 5	Tweet 6	Tweet 7	Tweet 8	Active Margin
Progress		19							19
Freedom	18	17	16		16				67
Intolerance							22	16	38
Equality	30		14			17			61
Empathy (absence of)								17	17
Ignorance				6			34		40
Misinformation				10			19		29
Scorn (harm)				14					14
Violence							17		17
Active margin	48	36	30	30	16	17	92	33	302

## Data Availability

The datasets used to generate the results for this study can be obtained from the authors when deemed necessary.
